# Self-monitoring of blood pressure among women with hypertensive disorders of pregnancy: a systematic review

**DOI:** 10.1186/s12884-022-04751-7

**Published:** 2022-05-31

**Authors:** Ping Teresa Yeh, Dong Keun Rhee, Caitlin Elizabeth Kennedy, Chloe A. Zera, Briana Lucido, Özge Tunçalp, Rodolfo Gomez Ponce de Leon, Manjulaa Narasimhan

**Affiliations:** 1 Department of International Health, Johns Hopkins Bloomberg School of Public Health, Baltimore, MD USA; 2 Department of Obstetrics and Gynecology, Harvard Medical Faculty Physicians, Boston, MA USA; 3 Department of Sexual and Reproductive Health and Research, World Health Organization, includes the UNDP/UNFPA/UNICEF/WHO/World Bank Special Programme of Research, Development and Research Training in Human Reproduction – HRP, 20 Avenue Appia, 1211, Geneva 27, Switzerland; 4 Latin American Center of Perinatology, Women and Reproductive Health PAHO/WHO, Montevideo, Uruguay

**Keywords:** Self-monitoring, Blood pressure, Hypertension, Pregnancy, Pre-eclampsia, Self-care

## Abstract

**Background:**

The World Health Organization (WHO) recommends self-monitoring of blood pressure (SMBP) for hypertension management. In addition, during the COVID-19 response, WHO guidance also recommends SMBP supported by health workers although more evidence is needed on whether SMBP of pregnant individuals with hypertension (gestational hypertension, chronic hypertension, or pre-eclampsia) may assist in early detection of pre-eclampsia, increase end-user autonomy and empowerment, and reduce health system burden. To expand the evidence base for WHO guideline on self-care interventions, we conducted a systematic review of SMBP during pregnancy on maternal and neonatal outcomes.

**Methods:**

We searched for publications that compared SMBP with clinic-based monitoring during antenatal care. We included studies measuring any of the following outcomes: maternal mortality, pre-eclampsia, long-term risk and complications, autonomy, HELLP syndrome, C-section, antenatal hospital admission, adverse pregnancy outcomes, device-related issues, follow-up care with appropriate management, mental health and well-being, social harms, stillbirth or perinatal death, birthweight/size for gestational age, and Apgar score. After abstract screening and full-text review, we extracted data using standardized forms and summarized findings. We also reviewed studies assessing values and preferences as well as costs of SMBP.

**Results:**

We identified 6 studies meeting inclusion criteria for the effectiveness of SMBP, 6 studies on values and preferences, and 1 study on costs. All were from high-income countries. Overall, when comparing SMBP with clinic-monitoring, there was no difference in the risks for most of the outcomes for which data were available, though there was some evidence of increased risk of C-section among pregnant women with chronic hypertension. Most end-users and providers supported SMBP, motivated by ease of use, convenience, self-empowerment and reduced anxiety. One study found SMBP would lower health sector costs.

**Conclusion:**

Limited evidence suggests that SMBP during pregnancy is feasible and acceptable, and generally associated with maternal and neonatal health outcomes similar to clinic-based monitoring. However, more research is needed in resource-limited settings.

**Systematic review registration:**

PROSPERO CRD42021233839.

## Background

Hypertensive disorders of pregnancy affect approximately 10% of pregnant individuals globally, and are among the leading causes of pregnancy-related mortality and morbidities for women, adolescent girls, and their newborns, particularly in low and middle-income countries (LMICs) [1–3]. Hypertension in pregnancy can also lead to long-term health conditions such as development of chronic hypertension and is associated with pre-eclampsia, which can result in a range of morbidities in newborns, including low birth weight and respiratory distress syndrome [4–6]. Early antihypertensive treatment and timely delivery can prevent morbidity and potentially mortality [7]. Improving management of hypertension during pregnancy is thus an essential aspect of quality care for maternal and neonatal health.

Despite many interventions implemented in LMICs to improve maternal and child health over the past several decades, unfavorable health outcomes persist [8, 9]. Increased access to and use of high-quality health care during pregnancy and childbirth remains a priority public health goal, and identifying cost-effective intervention strategies is critical in resource-limited settings [10]. Innovative strategies to improve antenatal care management, including through self-care interventions, have the potential to improve the health outcomes of pregnant individuals and their newborns [11].

Routine antenatal care contacts generally include blood pressure measurement, but blood pressure changes may be missed between contacts. Self-monitoring of blood pressure (SMBP), a strategy in which people take a more active role in their own health care by measuring their own blood pressure [12], may be particularly useful in settings where access to and resources for conventional antenatal care are limited. SMBP has also been referred to as home blood pressure monitoring (HBPM), which focuses on the setting instead of the individual taking the measurement. Among the general hypertensive population, the evidence for the efficacy and feasibility of HBPM has been associated with improved hypertension control compared to clinic-based monitoring [13–15], though its impact depends on the specific outcomes that were assessed [16]. A recent review found that SMBP had limited impact on blood pressure control in the general population, unless accompanied by certain co-interventions [17]. However, less is known about SMBP specifically for pregnant individuals and their newborns [18]. Two recent reviews reported mixed benefits of HBPM compared to clinic-based monitoring for multiple maternal and neonatal outcomes among pregnant and postpartum individuals [19, 20], suggesting that home-based monitoring may be as effective as receiving provider-administered care.

We conducted this systematic review in the context of expanding the evidence base of the WHO guidelines on self-care interventions for health [21], which include existing WHO recommendations on self-care interventions during pregnancy, childbirth and post-natal care [22, 23]. SMBP is recommended by WHO for the management of hypertension in appropriate patients where the affordability of the technology has been established [21, 24]. Self-monitoring of hypertensive disorders of pregnancy (including individuals with pregnancy-induced hypertension/gestational hypertension, chronic hypertension, or pre-eclampsia) has been identified as a priority topic for expanding the evidence-base on self-care interventions. Building upon WHO recommendations on the prevention and treatment of pre-eclampsia and eclampsia, this systematic review also included considerations for support to pregnant individuals during health emergencies such as the COVID-19 pandemic [25].

## Methods

This review addressed the following question: Should SMBP among individuals with hypertensive disorders of pregnancy be made available in addition to clinic check-ups? We reviewed the extant literature in three areas relevant to this question: (1) effectiveness of the intervention, (2) values and preferences of end-users and providers, and (3) cost information. We included all three of these areas because they are components of the evidence-based process used to inform WHO guideline development [26]. We followed Preferred Reporting Items for Systematic review and Meta-Analysis (PRISMA) guidelines [27] and registered the protocol on the International Prospective Register of Systematic Reviews (PROSPERO registration number CRD42021233839). Ethical approval was not required for this systematic review, since all data came from published articles.

### Effectiveness review inclusion criteria

We designed the effectiveness review according to PICO format as follows:

- **Population**: Pregnant individuals with hypertension (gestational hypertension, chronic hypertension, and pre-eclampsia)
- **Intervention**: Self-monitoring of blood pressure (either by the pregnant individual or by another layperson, such as a family member)
- **Comparison**: Clinic blood pressure monitoring by health care providers during antenatal care (ANC) contacts only
- **Outcomes**:
  - Maternal outcomes:
    1. Maternal mortality or near-miss
    2. Eclampsia or pre-eclampsia (for those without pre-eclampsia prior to entering the study)
    3. Long-term risk or complications: stroke, cardiovascular outcomes, chronic kidney (renal) disease, or chronic hypertension
    4. Autonomy (measured by self-efficacy, self-determination, empowerment)
    5. HELLP syndrome
    6. Cesarean section
    7. Antenatal hospital admission
    8. Adverse pregnancy outcomes: spontaneous abortion, premature rupture of membranes, placental abruption
    9. Device-related issues (e.g. test failure, problems with manufacturing, packaging, labelling, or instructions for use)
    10. Follow-up care with appropriate management
    11. Mental health and well-being (e.g. anxiety, stress, self-harm)
    12. Social harms: stigma, discrimination, intimate partner violence
  - Fetal/neonatal outcomes:
    1. Stillbirth or perinatal death
    2. Birthweight and size for gestational age
    3. Apgar score

To be included in the review, an article must have: (1) had a study design that compared SMBP among pregnant individuals with hypertension at home or outside the clinic setting to only during ANC by a health care provider, including randomized controlled trials, non-randomized controlled trials, and comparative observational studies (including prospective controlled cohort studies, retrospective controlled cohort studies, cross-sectional studies, controlled before-after studies and interrupted time series) that compare individuals who received the intervention to those who did not, (2) measured one or more of the above outcomes, and (3) been published in a peer-reviewed journal.

There are many different categories and definitions of hypertensive disorders in pregnancy, which vary across national medical societies and organizations [28]. For the purposes of this review, we included any study that presented data for pregnant individuals with hypertension, no matter how it was defined; however, we carefully reviewed definitions used to ensure comparability when comparing results across studies.

We defined SMBP as monitoring of blood pressure either by the pregnant individual or by another layperson, such as a family member, in which the measurement is initiated by the lay user (whether or not the sphygmanometer was automatic) and the blood pressure data is recorded and/or reviewable by the lay user prior to or at the same time as the provider (whether on paper or electronically). We excluded ambulatory blood pressure monitoring [29], wherein users typically wear a monitor on their upper arms for 24 hours of measurement, and telehealth [30], where end-users may not have the ability to independently record or review their blood pressure measurements without concurrent interaction with a health provider through a mobile or online app.

We did not restrict study inclusion on the basis of language or intervention location. Articles in English, French, Spanish, and Chinese were coded directly; articles in other languages were translated.

### Search strategy and screening

We searched four electronic databases (PubMed, CINAHL, LILACS and EMBASE) through the search date of November 9, 2020 using the following search string (designed for PubMed and adapted for each database):

(“blood pressure” [mesh] OR hypertension [mesh] OR “Blood Pressure Monitoring, Ambulatory” [mesh] OR hypertension [tiab] OR hypertensive [tiab] OR PIH [tiab] or “blood pressure” [tiab] OR pre-eclampsia [tiab] or bp [tiab])

AND

(pregnancy [Mesh] OR pregnancy [tiab] OR pregnant [tiab] OR peri-natal [tiab] OR perinatal [tiab] OR antenatal [tiab] OR maternal [tiab])

AND

(“self care”[Mesh] OR “self-care”[tiab] OR “self-monitoring”[tiab] OR “self-management”[tiab] OR “self-monitor”[tiab] OR “self-manage”[tiab] OR “self-monitored”[tiab] OR “self-managed”[tiab] OR “self-evaluation”[tiab] OR “self-test”[tiab] OR “self-testing”[tiab] OR “home”[tiab] OR “pharmacy”[tiab])

We further searched for ongoing randomized controlled trials (RCTs) through clinicaltrials.gov, the WHO International Clinical Trials Registry Platform, the Pan African Clinical Trials Registry, and the Australian New Zealand Clinical Trials Registry. In addition, we searched the Cochrane Database of Systematic Reviews for related reviews. Secondary reference searching was also conducted on all studies included in the review and relevant reviews. Finally, selected experts in the field were contacted to identify additional articles not identified through other search methods.

Titles, abstracts, citation information, and descriptor terms of citations identified through the search strategy were screened by a member of the study staff. Full-text articles were obtained of all selected abstracts, and two independent reviewers assessed all full-text articles for eligibility to determine final study selection. Differences were resolved through consensus.

### Data management and analysis

Two reviewers independently abstracted data using standardized forms. Differences in data extraction were resolved through consensus and referral to a senior study team member from WHO as necessary. We gathered the following information from each article: study identification (authors, type of citation, year of publication), study description (study objectives, location, population characteristics, type and definition of hypertension, type of blood pressure apparatus, individual taking the blood pressure at home, description of any additional intervention components such as any education, training, support provided, study design, sample size, follow-up periods, and loss to follow-up), and outcomes (analytic approach, outcome measures, comparison groups, effect sizes, confidence intervals, significance levels, conclusions, study limitations).

For RCTs, risk of bias was assessed using the Cochrane Collaboration’s tool for assessing risk of bias [31]. For studies that were not randomized trials but were comparative, study rigor was assessed using the Evidence Project 8-item checklist for intervention evaluations [32].

Data were analyzed according to coding categories and outcomes. Where there were multiple studies reporting the same outcome, meta-analysis was conducted using random-effects models to combine risk ratios (RRs) or mean differences (MDs) with the program Comprehensive Meta-Analysis (CMA). For each PICO outcome category, data were summarized in a GRADE Evidence Profile table using GRADEPro, prioritizing RCT data over observational data where available.

Where possible, we planned to stratify all analyses by the following categories/subgroups: form of hypertension during pregnancy (gestational hypertension, chronic hypertension, and pre-eclampsia), prior risk of hypertension, age (adolescent girls and young adults of ages 10–14, 15–19, and 15–24, and women of ages 25+), type of blood pressure (BP) monitor, vulnerabilities (i.e. obesity, poverty, disability, rural/urban, literacy/educational level), and high-income versus low or middle-income countries.

### Complementary reviews

We conducted complementary reviews to examine the values and preferences of end-users and providers and costs related to SMBP. We used the same search strategy to identify studies to be included in these reviews. These studies could have been qualitative or quantitative in nature, but had to present primary data collection; think pieces and review articles were not included. We summarized this literature qualitatively and organized findings by study design and methodology, location, and population.

#### Values and preferences review

We focused on studies examining the values and preferences of pregnant individuals who expressed willingness to self-manage their blood pressure (including non-hypertensive individuals) and were able to accurately record and report blood pressure measurements to their healthcare provider. Given the growing use of remote patient monitoring and web/app-based health services [33], we included studies involving telehealth. We also included studies examining the values and preferences of healthcare providers, including their willingness to trust their clients. We considered issues related to age of availability, informed decision-making, coercion and seeking redress in this section.

#### Cost review

We included studies in this review if they presented primary data comparing costing, cost-effectiveness, cost-utility, or cost-benefit of the intervention and comparison listed in the PICO question above, or if they presented cost-effectiveness of the intervention as it related to the PICO outcomes listed above. This included both cost to the health system and cost to the end-user. Cost literature was classified into four categories: health sector costs, other sector costs, patient/family costs, and productivity impacts.

### Patient and public involvement

Feedback on the review protocol and analysis was received from the WHO patient safety working group. Patients were involved in a global survey of values and preferences conducted to inform the WHO guideline on self-care interventions and play a role in the overall recommendation informed by this review.

## Results

**Fig. 1:**
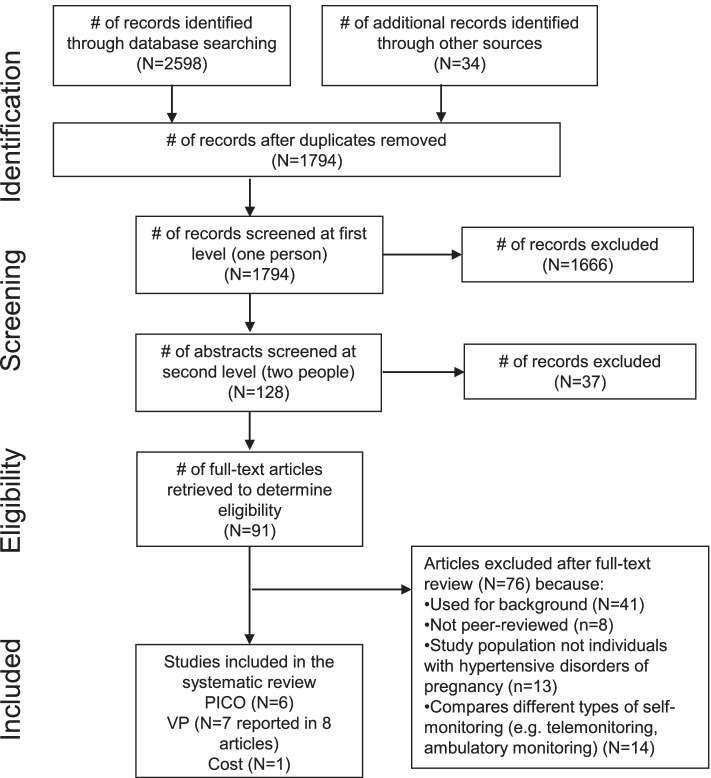
PRISMA flow chart showing disposition of citations through the search and screening process

Our database search yielded 2598 records, and we identified another 34 through hand- and secondary searching (Fig. 1). Of the 1794 unique records, we retained 91 for full-text review. Ultimately, we included 6 studies in the effectiveness review [34–39], 7 studies (reported in 8 articles) in the values and preferences review [40–49], and 1 study in the cost review [50].

### Effectiveness review

Overall, 6 studies met the inclusion criteria for the effectiveness review [34–39]. Table 1 presents descriptive data for the 1 RCT and 5 observational studies. To assess the highest-certainty evidence for each PICO outcome category, we included RCT data in the GRADE Evidence Profile (Table 2) when available, and where RCT data were not available, we included data from observational studies. Given the small number of studies presenting outcome data, no further stratifications from our a priori list were possible.

**Table 1:** Description of studies included in effectiveness review

Study	Location	Population	Study design and sampling	Intervention	Comparator
**Used in GRADE**					
Pealing et al., 2019 [37] (OPTIMUM-BP)	United Kingdom	Pregnant women with chronic or gestational hypertension without pre-eclampsia; authors did not report education or health literacy level of participants. *N* = 53 (self, chronic); 30 (provider, chronic); 49 (self, gestational); 22 (provider, gestational)	RCT Non-probability facility-based	Daily self-monitoring using an automated BP monitor (Microlife WatchBP) recorded on paper or submitted via text message or app; trained how to measure BP, symptoms of hypertensive disease in pregnancy, interpret readings using algorithm and when to contact provider – otherwise, received routine antenatal care.	Routine care at antenatal visits
Kalafat et al., 2019 [36]	United Kingdom: London	Pregnant women with gestational hypertension; authors did not report education or health literacy level of participants. *N* = 80 (self); 63 (provider)	Prospective cohort Non-probability facility-based	Daily self-monitoring using an automated BP monitor (Microlife WatchBP) recorded on paper or submitted via app; trained how to measure BP, interpret readings using algorithm and when to contact provider – otherwise, received routine antenatal care.	Routine care at prenatal visits
Perry et al., 2018 [38]	United Kingdom: London	Pregnant women with chronic hypertension, gestational hypertension, or high risk of developing pre-eclampsia; authors did not report education or health literacy level of participants. *N* = 108 (self); 58 (provider)	Case-control Non-probability facility-based	Daily self-monitoring using an automated BP monitor (Microlife WatchBP) recorded on paper or submitted via app; trained how to measure BP, interpret readings using algorithm and when to contact provider – otherwise, received routine antenatal care.	Routine care at prenatal visits
**Not used in GRADE because these observational studies reported the same outcomes as the RCT**					
Rayburn et al., 1985 [39]	United States: Ann Arbor	Pregnant women with chronic hypertension; authors did not report education or health literacy level of participants. *N* = 33	Multi-cohort (prospective cohort for SMBP), retrospective cohort for provider) Non-probability facility-based	Daily twice-a-day self-monitoring using a BP kit recorded on paper; trained how to measure BP, interpret readings and when to contact provider – otherwise, received routine antenatal care.	Historical group of women with chronic hypertension who received routine care at prenatal visits
Fukushima et al., 2002 [34]	United States: Los Angeles	Pregnant women with pregnancy-induced hypertension. Women in the self-monitoring group were identified to be at most-risk by hypertension severity. Authors did not report education or health literacy level of participants. *N* = 19 (self); 180 (provider)	Prospective cohort Non-probability facility-based	Daily self-monitoring for several times a day at home using a system uniquely developed to measure cardiovascular dynamics data for this study, installation and user training for which were provided, with the women transmitting the results via facsimile equipment – otherwise, received routine antenatal care.	Routine care at prenatal visits
Iwama et al., 2016 [35]	Japan: Sendai	Pregnant women including those with chronic hypertension but did not use antihypertensive drugs before 20 weeks’ gestation. Authors did not report education or health literacy level of participants. *N* = 605 (self-monitoring performed within a week of clinic-based monitoring instance by all women)	Prospective cohort Non-probability facility-based	At least one instance of BP self-monitoring within a week of clinic-based measurement using semi-automatic BP monitor (Omron HEM-747IC or HEM-7080IC)	One-time measurement at the clinic between using semi-automatic BP monitor (Omron HEM705IT)

**Table 2:** GRADE Evidence Profile

Certainty assessment							№ of patients		Effect		Certainty	Importance
№ of studies	Study design	Risk of bias	Inconsistency	Indirectness	Imprecision	Other considerations	self-monitoring of blood pressure	clinic blood pressure monitoring	Relative (95% CI)	Absolute (95% CI)		
***RCT: MATERNAL: Eclampsia or pre-eclampsia (for those without pre-eclampsia prior to entering the study)***												
MATERNAL: Pre-eclampsia (among pregnant individuals with chronic hypertension)												
1 [37]	randomised trials	not serious ^a^	not serious ^b^	not serious	very serious ^c,d^	none	19/53 (35.8%)	5/30 (16.7%)	**RR 2.15** (0.89 to 5.17)	**192 more per 1000** (from 18 fewer to 695 more)	⨁⨁◯◯ LOW	CRITICAL
MATERNAL: Pre-eclampsia (among pregnant individuals with gestational hypertension)												
1 [37]	randomised trials	not serious ^a^	not serious ^b^	not serious	very serious ^c,d^	none	15/49 (30.6%)	8/22 (36.4%)	**RR 0.84** (0.42 to 1.69)	**58 fewer per 1000** (from 211 fewer to 251 more)	⨁⨁◯◯ LOW	CRITICAL
***RCT: MATERNAL: C-section***												
MATERNAL: C-section, total (among pregnant individuals with chronic hypertension) (assessed with: combining emergency pre-labor c-section, emergency c-section in labor, and elective pre-labor c-section)												
1 [37]	randomised trials	not serious ^a^	not serious ^b^	not serious	serious ^d,g^	none	39/53 (73.6%)	11/30 (36.7%)	**RR 2.01** (1.22 to 3.30)	**370 more per 1000** (from 81 more to 843 more)	⨁⨁⨁◯ MODERATE	IMPORTANT
MATERNAL: C-section, total (among pregnant individuals with gestational hypertension) (assessed with: combining emergency pre-labor c-section, emergency c-section in labor, and elective pre-labor c-section)												
1 [37]	randomised trials	not serious ^a^	not serious ^b^	not serious	very serious ^c,d,h^	none	25/49 (51.0%)	13/22 (59.1%)	**RR 0.86** (0.55 to 1.34)	**83 fewer per 1000** (from 266 fewer to 201 more)	⨁⨁◯◯ LOW	IMPORTANT
***RCT: MATERNAL: Antenatal hospital admission***												
MATERNAL: Admitted to intensive therapy unit (among pregnant individuals with chronic hypertension)												
1 [37]	randomised trials	not serious ^a^	not serious ^b^	not serious	very serious ^e^	none	0/53 (0.0%)	0/30 (0.0%)	not estimable		⨁⨁◯◯ LOW	CRITICAL
MATERNAL: Admitted to intensive therapy unit (among pregnant individuals with gestational hypertension)												
1 [37]	randomised trials	not serious ^a^	not serious ^b^	not serious	serious ^f^	none	1/49 (2.0%)	0/22 (0.0%)	not estimable		⨁⨁⨁◯ MODERATE	CRITICAL
***OBSERVATIONAL: MATERNAL: Adverse pregnancy outcomes: spontaneous abortion, premature rupture of membranes, placental abruption***												
MATERNAL: Composite maternal adverse outcomes (among pregnant individuals with gestational hypertension) (assessed with: acute renal failure (maternal serum creatinine level > 100 μmol/L antenatally or > 130 μmol/L postnatally) or need for dialysis, acute myocardial ischemia, need for third intravenous agent to control blood pressure (i.e. in addition to labetalol and hydralazine), hypertensive encephalopathy (altered mental status with characteristic cerebral imaging), cortical blindness, retinal detachment, stroke (ischemic or hemorrhagic), pulmonary edema or adult respiratory distress syndrome (defined by characteristic pulmonary imaging in addition to oxygen requirement), need for mechanical ventilatory support (other than for Cesarean section), disseminated intravascular coagulation, thrombotic thrombocytopenic purpura or hemolytic uremic syndrome, acute fatty liver, liver hematoma or rupture, placental abruption, and maternal death)												
1 [36]	observational studies	not serious	not serious ^b^	not serious	serious ^f^	none	1/80 (1.3%)	1/63 (1.6%)	**RR 0.79** (0.05 to 12.34)	**3 fewer per 1000** (from 15 fewer to 180 more)	⨁◯◯◯ VERY LOW	CRITICAL
MATERNAL: Composite maternal adverse outcomes (among pregnant individuals with chronic hypertension, gestational hypertension, or high risk of developing preeclampsia) (assessed with: acute renal failure (maternal serum creatinine level > 100 μmol/L antenatally or > 130 μmol/L postnatally) or need for dialysis, acute myocardial ischemia, need for third intravenous agent to control blood pressure (i.e. in addition to labetalol and hydralazine), hypertensive encephalopathy (altered mental status with characteristic cerebral imaging), cortical blindness, retinal detachment, stroke (ischemic or hemorrhagic), pulmonary edema or adult respiratory distress syndrome (defined by characteristic pulmonary imaging in addition to oxygen requirement), need for mechanical ventilatory support (other than for Cesarean section), disseminated intravascular coagulation, thrombotic thrombocytopenic purpura or hemolytic uremic syndrome, acute fatty liver, liver hematoma or rupture, placental abruption, and maternal death)												
1 [38]	observational studies	not serious	not serious ^b^	not serious	serious ^f^	none	1/108 (0.9%)	2/58 (3.4%)	**RR 0.27** (0.02 to 2.90)	**25 fewer per 1000** (from 34 fewer to 66 more)	⨁◯◯◯ VERY LOW	CRITICAL
***RCT: FETAL/NEWBORN: Stillbirth or perinatal death***												
FETAL/NEWBORN: Stillbirth or neonatal death (among pregnant individuals with chronic hypertension)												
1 [37]	randomised trials	not serious ^a^	not serious ^b^	not serious	serious ^f^	none	3/53 (5.7%)	0/30 (0.0%)	not estimable		⨁⨁⨁◯ MODERATE	CRITICAL
FETAL/NEWBORN: Stillbirth or neonatal death (among pregnant individuals with gestational hypertension)												
1 [37]	randomised trials	not serious ^a^	not serious ^b^	not serious	very serious ^e^	none	0/49 (0.0%)	0/22 (0.0%)	not estimable		⨁⨁◯◯ LOW	CRITICAL
***RCT: FETAL/NEWBORN: birthweight / size for gestational age***												
FETAL/NEWBORN: Birthweight in grams (among pregnant individuals with chronic hypertension)												
1 [37]	randomised trials	not serious ^a^	not serious ^b^	not serious	very serious ^d,i^	none	53	30	MD **300.2 g lower** (690.7 lower to 90.2 higher)		⨁⨁◯◯ LOW	CRITICAL
FETAL/NEWBORN: Birthweight in grams (among pregnant individuals with gestational hypertension)												
1 [37]	randomised trials	not serious ^a^	not serious ^b^	not serious	very serious ^d,i^	none	49	22	MD **54.2 g higher** (341.7 lower to 450 higher)		⨁⨁◯◯ LOW	CRITICAL
FETAL/NEWBORN: Small for gestational age (among pregnant individuals with chronic hypertension) (assessed with: birthweight < 10th percentile)												
1 [37]	randomised trials	not serious ^a^	not serious ^b^	not serious	very serious ^c,d,j^	none	8/53 (15.1%)	1/30 (3.3%)	**RR 4.53** (0.59 to 34.48)	**118 more per 1000** (from 14 fewer to 1000 more)	⨁⨁◯◯ LOW	CRITICAL
FETAL/NEWBORN: Small for gestational age (among pregnant individuals with gestational hypertension) (assessed with: birthweight < 10th percentile)												
1 [37]	randomised trials	not serious ^a^	not serious ^b^	not serious	very serious ^c,d,k^	none	9/49 (18.4%)	4/22 (18.2%)	**RR 1.01** (0.35 to 2.93)	**2 more per 1000** (from 118 fewer to 351 more)	⨁⨁◯◯ LOW	CRITICAL

*CI* Confidence interval, *RR* Risk ratio, *MD* Mean difference

^a^Risk of bias: Not downgraded for detection bias. Participant and provider blinding was not possible given the nature of the intervention. Detection bias was unlikely as the outcome unlikely to have been affected by lack of blinding

^b^Inconsistency: This could not be evaluated, as there is only a single study

^c^Imprecision: Downgraded because 95% CI for RR includes both 1 (no effect) AND either appreciable harm (0.75) or appreciable benefit (1.25)

^d^Imprecision: Downgraded for very small sample size

^e^Imprecision: Downgraded twice for non-existent event (in both arms) and very small sample size

^f^Imprecision: Downgraded for very rare event and very small sample size

^g^Pealing et al. 2019 also disaggregates data comparing between SMBP and clinic monitoring for different types of c-section (maternal outcome of interest) among women with chronic hypertension. Number (%) in the SBMP vs Usual care group respectively: elective pre-labor c-section: 15 (28%) vs 4 (13%); emergency c-section in labor: 10 (19%) vs 2 (7%); elective pre-labor c-section: 14 (26%) vs 5 (17%)

^h^Pealing et al. 2019 also disaggregates data comparing between SMBP and clinic monitoring for different types of c-section (maternal outcome of interest) among women with gestational hypertension. Number (%) in the SBMP vs Usual care group respectively: elective pre-labor c-section: 4 (8%) vs 5 (23%); emergency c-section in labor: 11 (22%) vs 1 (4%); elective pre-labor c-section: 9 (18%) vs 3 (14%)

^i^Imprecision: Downgraded because 95% CI for mean difference includes a range of about 800 g (infants are classified as low birthweight at 2500 g)

^j^Pealing et al. 2019 also presents data comparing between SMBP and clinic monitoring for another measure of birthweight (neonatal outcome of interest) among women with chronic hypertension. Number (%) in the SBMP vs Usual care group respectively for birthweight <3rd centile: 2 (4%) vs 1 (3%)

^k^Pealing et al. 2019 also presents data comparing between SMBP and clinic monitoring for another measure of birthweight (neonatal outcome of interest) among women with gestational hypertension. Number (%) in the SBMP vs Usual care group respectively for birthweight <3rd centile: 2 (4%) vs 0 (0%)

One small feasibility RCT among 154 pregnant women with chronic or gestational hypertension without pre-eclampsia at four health centers in the UK (OPTIMUM-BP) compared SMBP with usual care from December 2015 to December 2017 [37]. This study was classified as low risk of bias for all outcomes; though participant and provider blinding was not possible given the nature of the intervention (potential detection bias), the clinical outcomes measured by this trial were unlikely to have been affected by lack of blinding. This RCT reported on several maternal and neonatal outcomes of interest: pre-eclampsia, c-section, antenatal hospital admission, stillbirth or perinatal death, and birthweight/size for gestational age.

Two observational studies at the same hospital in the UK, one case-control among pregnant women with gestational hypertension [38] and one prospective cohort among pregnant women with chronic hypertension, gestational hypertension, or high risk of developing pre-eclampsia [36], examined maternal adverse events, comparing between SBMP and routine ANC. These studies were also judged to have low risk of bias. Three additional observational studies were not included in the GRADE Evidence Profile because they reported the same outcomes as the RCT [34, 35, 39].

### Eclampsia or pre-eclampsia

Comparing between individuals who self-monitored blood pressure and those who had their BP measured during routine ANC, the single RCT found no statistically significant difference in pre-eclampsia rate among pregnant individuals with either chronic (RR: 2.15, 95% confidence interval (CI): 0.89–5.17) or gestational (RR: 0.84, 95% CI: 0.42–1.69) hypertension [37]. This was graded as low-certainty evidence because of the small sample size and because the 95% confidence interval for relative risk crossed 1 and included the potential for both appreciable benefit or appreciable harm.

### Cesarean section

The RCT found that SMBP was associated with higher C-section rates among pregnant individuals with chronic hypertension compared to clinic monitoring (RR: 2.01, 95% CI: 1.22–3.30, moderate certainty – downgraded for very small sample size), but there was no difference between SMBP and clinic monitoring among participants with gestational hypertension (RR: 0.86, 95% CI: 0.55–1.34) [37]. This was graded as low-certainty evidence for the same reasons as the previous outcome.

### Antenatal hospital admission

The RCT found no difference between SMBP and clinic monitoring on antenatal hospital admissions, for pregnant individuals with either chronic or gestational hypertension. This was considered low-to-moderate certainty evidence because of the very small sample size and very rare or non-existent events (in some groups, there were no records of admission to the hospital inpatient intensive therapy unit) [37].

### Adverse pregnancy outcomes

Two observational studies found no difference between SMBP and clinic monitoring on maternal morbidity, as measured with composite maternal adverse outcomes among pregnant individuals with gestational hypertension (RR: 0.79, 95% CI: 0.05–12.34) [36] or among pregnant individuals with chronic hypertension, gestational hypertension, or high risk of developing preeclampsia (RR: 0.27, 95% CI: 0.02–2.90) [38]. This was graded as very low certainty evidence because the number of events in both groups was very small (very rare events, despite reporting a composite outcome of many types of maternal adverse events combined) and because of the very small sample size.

### Stillbirth or perinatal death

The RCT showed no difference between SMBP and clinic monitoring of BP on stillbirth or perinatal death. This was considered low-to-moderate certainty evidence because of the very small sample size and very rare (or non-existent) events in one or both arms [37].

### Birthweight / size for gestational age

The RCT showed that SMBP was associated with lower birthweight (MD: -300.2, 95% CI: − 690.7-90.2) and a higher rate of infants being born small for gestational age (RR: 4.53, 95% CI: 0.59–34.48) among pregnant individuals with chronic hypertension compared with clinic monitoring, though these associations were not statistically significant [37]. The associations were similarly non-significant for those with gestational hypertension (MD in birthweight: 54.2, 95% CI: -341.7-450; RR for being born small for gestational age: 1.01, 95% CI: 0.35–2.93) [37]. This was graded as low-certainty evidence for the same reasons as the pre-eclampsia outcome.

### Other outcomes of interest

No quantitative comparative data were identified from either RCTs or observational studies related to maternal mortality or near-miss; long-term term risk or complications (e.g. stroke, cardiovascular outcomes, chronic kidney (renal) disease, or chronic hypertension); autonomy (measured by self-efficacy, self-determination, empowerment); HELLP syndrome; device-related issues; follow-up care with appropriate management; mental health and wellbeing (e.g. anxiety, stress, self-harm); social harms (e.g. stigma, discrimination, intimate partner violence); or Apgar score.

### Values and preferences review

Seven studies reported in 8 articles [40–47] conducted in North America, Europe, and Oceania (all high-income countries) provided values and preferences data on SMBP for pregnant individuals with hypertensive disorders (Table 3). Two of these studies also reported data on providers’ perspectives [40, 46]. In terms of study design, 4 were quantitative studies, 3 were qualitative, and 1 was mixed methods; all employed non-probability facility-based sampling methods for participant recruitment.

**Table 3:** Description of studies presenting values and preferences data

Study	Location	Population and sample size	Data collection method	Self-monitoring description
Hinton et al., 2017 [41] (BuMP)	United Kingdom	Pregnant women with elevated risk of hypertension or pre-eclampsia including a family history, advanced age, high BMI, and renal disease. End-user qualifications (within the British educational system) ranged from first degree (*n* = 7), GCSE 0 Level or CSE (*n* = 1), Professional qualification (*n* = 3), post-graduate or above (*n* = 3), and unknown (*n* = 1). *N* = 15	Semi-structured interview	Self-monitoring blood pressure using an automated electronic sphygmomanometer validated for use in pregnancy and preeclampsia (Microlife WatchBP home). Two measurements following 5 minutes of rest were required in the morning and evening, taking place on Monday, Wednesday, and Friday of the week. Self-monitoring also involved receiving feedback on their hypertension status with appropriate follow-up measures, and enabled submitting the blood pressure measurements to the central server. Women were enrolled at 12–16 weeks in their pregnancy and were instructed to self-monitor for up to 6 weeks post-partum.
Hinton et al., 2020 [40]	United Kingdom	Obstetricians, hospital and community midwives, pharmacists, and physicians-in-training. *N* = 147	Semi-structured interview, focus groups	N/A (providers reported their views on various topics surrounding the patients’ self-monitoring of blood pressure during pregnancy)
Jongsma et al., 2020 [42] van den Heuvel et al., 2020a [46, 47] (SAFE@HOME)	Netherlands	Pregnant women with singleton pregnancy and at least one of the predetermined risk factors of preeclampsia including chronic hypertension and maternal cardiac or renal diseases. Participants had to have a “good understanding of either the Dutch or English language”, but authors provided no further report of educational or health literacy level. *N* = 103 invited (additional 133 as a retrospective control sample for van den Heuvel 2020)	Validated questionnaires, semi-structured interviews based on the van den Heuvel study sample (Jongsma 2020) Surveys in the context of a case control study (van den Heuvel 2020)	Self-monitoring blood pressure using an automated sphygmomanometer (iHealth Track). Patients were to submit a single measurement on every weekday before 10 AM, which was transmitted to the application on phone and could be manually checked by patients prior to forwarding it to the platform. Self-monitoring also involved receiving feedback on their hypertension status and symptoms with appropriate follow-up measures. Self-monitoring began from 16 weeks of gestation until delivery.
Marko et al., 2016 [43] (Babyscripts)	United States	Pregnant women of ages between 18 and 40 years with self-reported low-risk pregnancy status. Authors did not report education or health literacy level of participants. *N* = 8	Surveys in the context of a prospective observational study	Self-monitoring blood pressure using an automated electronic sphygmomanometer (Wireless Blood Pressure Monitor, Withings). Measurements were collected on a weekly basis, which were transmitted to the Babyscripts application installed on a mobile phone. These were available for review by both the patient and provider. Self-monitoring also involved receiving feedback on their blood pressure goals and alerting the provider. Women were enrolled at 8–10 weeks of gestation and followed until delivery.
Sheehan et al., 2019 [44]	United Kingdom	Women of 30–41 years of age with hypertensive disorders of pregnancy. Authors did not report education or health literacy level of participants. *N* = 8	Semi-structured interviews	Self-monitoring blood pressure at home using a validated blood pressure monitor on a daily basis. Measurements were submitted on the phone application. Self-monitoring also involved answering questions for signs of pre-eclampsia. Women completed at least 8 weeks of self-monitoring of pressure at home.
Taylor et al., 2001 [45]	New Zealand	A combination of two separate groups consisting of healthy pregnant women (*N* = 120) and inpatient women with preeclampsia (*N* = 40). Authors did not report education or health literacy level of participants.	Questionnaires	Self-monitoring using Omron HEM-705CP monitor on four occasions at home over 24 hours. For inpatient women with preeclampsia who had severe hypertension, additional measurements were collected. No feedback on blood pressure measurement was provided to women.
van den Heuvel et al., 2020b [46]	Netherlands	Obstetrics care professionals affiliated with hospitals that had pregnancy and/or childbirth care departments in the Netherlands *N* = 57	Web-based survey	Self-monitoring blood pressure as part of daily pregnancy monitoring, with the help of hospital personnel traveling to the pregnant individuals’ homes or the help of devices used by the pregnant individuals at home in the absence of hospital personnel

Overall, most end-users found SMBP highly satisfactory or acceptable. In fact, when end-users were specifically asked whether they would recommend SMBP to others in the SAFE@HOME study, almost all (especially multiparous women) reported that they would [42, 47]. For end-users, reasons for liking SMBP included the technical ease of use [43] and convenience of the device while conducting daily activities [41, 42]. There was some variation in end-users’ perception of ease of use; one study found that a certain brand and model of the device used for SMBP was perceived to be uncomfortable and noisy [45].

Reasons for liking SMBP went beyond the technical qualities of the self-monitoring device. For many end-users, SMBP reduced anxiety about their health during pregnancy; those who reported history of preeclampsia felt that self-monitoring allowed them to have more accurate and up-to-date information on their own health [41]. Women also reported feeling reassured when the SMBP device confirmed their BP status as “normal” [42]. In addition, SMBP was seen as helpful for encouraging healthy pregnancy-related behaviors [43]. End-users widely agreed that SMBP was beneficial and conducive to decreased stress during pregnancy, and they appreciated the reduction in frequency of care visits [42, 44].

End-users also noted SMBP for its role in facilitating self-empowerment. The practice of SMBP created the impression that they were taking a greater role in self-care as related to blood pressure, pregnancy, and health [42] through taking initiative in using the device [45], and the resulting sense of empowerment helped to alleviate anxiety [44]. Despite SMBP reducing the number of care visits, many patients whose SMBP devices enabled them to communicate with their providers (i.e. through apps for remote monitoring and telehealth) felt even more connected to their care team [43]. Generally, women agreed that they would continue to use SMBP themselves and recommend others to do the same [42, 47].

Two studies in the Netherlands and the UK reported values and preferences surrounding SBMP from the provider perspective [40, 41, 46]. Healthcare providers generally approved of SMBP, though with several concerns. The most frequently mentioned advantages of this strategy were improved patient comfort and reductions in emotional burden of admission, as well as better rest/less stress, increased patient autonomy, satisfaction, and safety, and decreased over-medicalization during pregnancy. However, clinicians stressed that the practice may be vulnerable to inaccurate and unreliable BP measurements, whether related to technical issues or inability to follow instructions/conduct monitoring at home, leading to a false sense of security, and that end-users should be educated on how to responsibly react to abnormal BP. Some expressed worries about device security issues. Moreover, clinicians opined that SMBP may not be popular for care teams since self-monitoring would shift the responsibility for healthcare to individuals, which may lead to delays in providing care during emergencies or acute problems, curtailed direct communication/patient assessments with the consulting gynecologist, or other cost/reimbursement issues.

### Cost review

One study in the UK assessed the cost-minimization of HBPM (likely performed by the end-user at home, but not specified by the study authors) among hypertensive pregnant women using an automated BP machine linked to paper notes or smartphone app [50]. In terms of direct costs for the health system, authors found a mean cost savings per week per patient using HBPM compared with traditional BP monitoring at maternity outpatient hospital visits was £200.69, which increased to £286.53 when using a smartphone application instead of a diary to record the blood pressure readings and clinical symptoms, and receive feedbacks. When using process modeling for the health system, they predicted a weekly savings of £98.32–£245.80 per patient, depending on the number of outpatient visits (visit cost included: midwife, doctor, blood tests, and fetal cardiotocography). In a second modeling scenario, if hospital admission were needed to initiate treatment, costs were similar for HBPM and traditional monitoring, but these incidents were anticipated and modeled to be infrequent occurrences, leading to significant cost savings.

## Discussion

Compared with clinic BP monitoring, SMBP was associated with twice the rate of C-section among individuals with chronic hypertension but no difference in C-section among individuals with gestational hypertension. However, the overall certainty of this evidence is low, due to small sample size, few events, and wide CIs, so it remains unclear whether SMBP changes the risk of C-section compared with BP monitoring during routine ANC visits. No other associations between SMBP and outcomes of interest were observed for pregnant individuals with any type of hypertension (chronic hypertension, gestational hypertension, or high risk of pre-eclampsia). Most end-users found SMBP highly satisfactory or acceptable and cited various factors including the device’s ease of use, convenience, and ability to help them feel empowered as reasons for liking self-monitoring. SMBP was also found to incur significant cost savings compared to usual care, due in part to fewer clinic visits.

Insufficient research on SMBP among pregnant individuals with hypertension has been conducted in LMICs. All our included studies were conducted in high-income countries. However, studying the health implications of SMBP for pregnant women with hypertensive disorders is of particular importance in LMICs. Pre-eclampsia is the world’s second leading cause of direct maternal death [51], and management of hypertension in LMICs could be improved [52]. In resource-limited settings, including in humanitarian crises or during pandemics like COVID-19, workable strategies for prevention, maintenance, and early intervention are needed for pregnant individuals [51].

A 2020 Cochrane review examining settings and techniques for monitoring blood pressure during pregnancy [18] identified the same feasibility RCT [37] we did. Another review found that HBPM significantly reduced the odds of preeclampsia and prenatal hospital admission relative to clinic-based monitoring for pregnant women [20]. The differences between these reviews’ findings and ours are likely due to differences in the characteristics of the study populations: our review was restricted to pregnant women with hypertension, while these reviews included all pregnant women.

Multiple strategies for blood pressure monitoring exist, including ambulatory blood pressure monitoring (ABPM) or recording a person’s blood pressure at different intervals over a 24-hour period [29]. A recent RCT found no statistically significantly difference in blood pressure measurements between 24-hour ABPM and clinic-monitoring [53], suggesting that the former may be just as effective as the latter. However, this model of blood pressure monitoring may be more feasible in high-income countries, given the infrastructure needed to support remote/mobile monitoring from home, such as internet access or publicly accessible Bluetooth and Wireless connections [54]. A recent review found ABPM utilization in just 36% of LMICs [55]. Potential reasons for low uptake include rejection by end-users because of the device disrupting sleep, higher initial cost versus other modes of SMBP like automated sphygmomanometers, end-users having to overcome the burden of transporting the devices, and the lack of formal training for clinicians with ABPM in many settings [55].

A viable alternative is SMBP, with or without the support of a mobile health (mHealth) or telehealth component. Hodgkinson et al. recently published a review on the optimal SMBP schedule [56]. mHealth is already widely used in the high-income countries (e.g. smartphone applications) and is gaining pace in LMICs (e.g. SMS) for its utility in managing a variety of conditions, providing clinical support to patients, and easing health system burden by preventing unnecessary clinic visits [30, 57]. With high and growing mobile phone ownership globally, mHealth-based SMBP has the potential to be an effective, acceptable, and cost-saving tool for monitoring hypertension [58, 59] and improving obstetric outcomes [60].

The growth of the digital health field – and technology’s role in health – may provide opportunities to expand the use of certain SMBP approaches, especially in low-resource settings. WHO has made progress in classifying the various types of digital health interventions, one of which is personal health tracking including self-monitoring of health or diagnostic data by clients. While some innovative technologies may be more suitable for low-resource settings [61], future work should continue to investigate which digital technologies can facilitate accurate and more consistent BP measurements in LMICs. In an effort to build capacity and support to countries to use digital technologies for the delivery of evidence-based healthcare and health practices, WHO has started producing guidelines to facilitate the implementation of WHO guidelines and recommendations in the digital age [62].

Furthermore, there are several types of devices that can be used to measure BP. These devices can be manual/analogue or automated/electric, and each type of device presents a range of benefits and challenges for health workers as well as lay people. In general, manual devices are not recommended and are being phased out because of environmental concerns, need for frequent calibration to maintain measurement accuracy, and inaccurate BP measurements. Instead, automated devices are preferred and recommended, as they may produce more accurate and consistent measurements [29]. In all settings, good-quality, validated devices need to be available to provide accurate BP measurements. However, low-resource settings may face particular challenges with acquiring, properly maintaining and calibrating these devices, and ensuring their appropriate use.

This review has a number of strengths. We conducted a comprehensive search across multiple databases as well as a hand search and secondary search. We also rigorously assessed the methodological quality of studies and examined not only the effectiveness of SMBP compared to monitoring during ANC but also its acceptance by pregnant individuals and providers and costing. Through this process, we found that SMBP is generally not associated with poorer maternal or neonatal health outcomes, that pregnant individuals and providers generally supported use of SMBP, and that SMBP could be cost-saving for a health system.

This review also had several limitations. First, our definition of SMBP was very specific. We excluded ambulatory monitoring and remote/telemonitoring from our effectiveness review because these methods put the primary responsibility for health monitoring on the provider, bypassing the person-centered focus of self-care. Second, the evidence base for our effectiveness, values and preferences, and cost reviews was limited: small sample sizes precluded our ability to reveal any effect on SMBP on maternal and perinatal outcomes of interest like stillbirth, and our findings came exclusively from high-income settings. Future research should examine the ramifications of implementing SMBP in resource-limited settings. Included studies did not provide details on the health literacy assessment of participants, though a few mentioned educational level or general language competency, so further research could investigate the feasibility, acceptability, and impact of this strategy for end-users of varying health literacy levels. Of note, the included studies all occurred prior to significant changes in prenatal care delivery during the COVID-19 pandemic. It is possible that in the context of increased familiarity with both remote monitoring and self-management of chronic disease [63], providers and patients would have different attitudes, knowledge and potential use cases for SMBP during pregnancy. Finally, the included studies do not specifically address potential benefits of SMBP postpartum. A current focus of postpartum quality improvement efforts in the US includes improved ascertainment and treatment of hypertension after delivery [64]. We were unable to assess the impact of SMBP on outcomes after the delivery such as morbidity, hospital readmission, and unplanned care utilization.

Using the evidence from this review and discussion among the guideline development group, the WHO Consolidated guideline on self-care interventions for health and well-being published in 2021 included the following recommendation: “WHO suggests making the self-monitoring of blood pressure during pregnancy available as an additional option to clinic blood pressure monitoring by health workers during antenatal contacts only, for individuals with hypertensive disorders of pregnancy. (*Conditional recommendation; very low certainty evidence*)” [65].

## Conclusion

SMBP is commonly available, especially in high-income settings, and generally accepted by end-users and health workers, suggesting its feasibility as an additional option for monitoring blood pressure during the antenatal period for pregnant individuals with hypertension. This review of the existing limited literature suggests that SMBP can be a cost-effective approach to expanding health services to the end-user with similar outcomes as receiving typical care from ANC, but more research is needed in low-resource settings.

## Data Availability

Extracted data are available on request to the corresponding author. No additional data beyond what is presented in this article.

## Abbreviations

SMBP: Self-monitoring of blood pressure
WHO: World Health Organization
LMIC: Low and middle-income country
HBPM: Home blood pressure monitoring
PRISMA: Preferred Reporting Items for Systematic review and Meta-Analysis
RCT: Randomized controlled trial
RR: Risk ratios
MD: Mean difference
CMA: Comprehensive Meta-Analysis
BP: Blood pressure
SMART: Standards-based, machine-readable, adaptive, requirements-based, and testable
